# Correlation between Rs2108622 Locus of CYP4F2 Gene Single Nucleotide Polymorphism and Warfarin Dosage in Iranian Cardiovascular Patients

**Published:** 2017

**Authors:** Shahdah Khosropanah, Seyed Nooreddin Faraji, Hamzeh Habibi, Majid Yavarian, Roohollah Mansoori, Sezaneh Haghpanah

**Affiliations:** a *School of Medicine, Shiraz University of medical science, Shiraz, Iran. *; b *School of Advanced Medical Sciences and Technologies, Shiraz University of Medical Sciences, Shiraz, Iran.*; c *Namazi hospital, Shiraz University of Medical Sciences, Shiraz, Iran.*

**Keywords:** rs2108622, CYP4F2, Polymorphism, Warfarin, Cardiovascular disease

## Abstract

Many cardiovascular diseases may require lifelong anticoagulation therapy. Warfarin is the most prescribed medication in this regard with serious side effects in some patients. Several single nucleotide polymorphisms (SNPs) affecting cytochrome P450 system can impact on warfarin metabolism and dosing. 230 cardiovascular patients have participated in the study. The INR levels were 1.5 to 3.5 with a mean range of 2.8. The subjects were divided into two case and control groups. The rs2108622 SNP of the CYP4F2 gene and its effect on warfarin dose requirements in these patients was evaluated.The results of our study showed a correlation between age and warfarin dosage. The overall frequency of the CC and TT allele of rs2108622 was 53.1% and 18.6%. Daily average dose of warfarin in CC, CT and TT variants was 3.5 ± 1.6, 4.5 ± 2.1 and 5.3 ± 2.1 respectively. The daily warfarin dose in patients with CC allele was significantly lower than that for CT or TT. The patients with TT allele required a 1.8 mg/day higher dose of warfarin than that of CC.While there are many studies regarding relation of age and warfarin dose, however, there are contradictory results about pharmacogentic status and warfarin dose in different ethnics. Our study demonstrates that polymorphism in the CYP4F2 rs2108622 has a significant impact on the warfarin requirements in Iranian patients.

## Introduction

Since 1950s that warfarin was used as an oral anticoagulant medication, yet it is the only oral anticoagulant approved by the US Food and Drug Administration (1). Use of warfarin is still increasing so that during 1998 to 2004 years, its prescription has increased to 45% (2). Warfarin has many applications such as prevention of thromboembolic events, pulmonary embolism and stroke, coronary malfunction, atrial fibrillation, and in prosthetic valve placements (3-5). However, according to the FDAʹs Adverse Event Reporting System, it is one of the medications with highest serious side effects among the top 10 drugs especially in its initial phase of treatment (2, 6). While several warfarin initiation or extended protocols such as “triple oral antithrombotic therapy” and “pharmacist-managed protocol” have been suggested and developed to reduce the risk of the medication; however, the results show considerable uncertainty for appropriate warfarin dosage. This does variety is due to inter individual and interethnic differences; so, physicians have some challenges for initiation of the therapy yet (1, 7-12). If warfarin is not controlled carefully by monitoring international normalized ratio (INR) it may cause necrosis (13),osteoporosis (14), increased risk of bleeding (15), and consequence side effects due to the hemorrhage such as purple toe syndrome (16). In general, dosing of warfarin is complicated because it has a narrow therapeutic window and also several parameters can affect it such as age, Body mass index (BMI) >30, gender, conditions for warfarin indication, interaction with other medications and certain foods, race, genetic, etc. (17-21). Between these, one of the first and main problems is interaction with many commonly used medications and certain foods(19). The second main problem recently considered in dosing of warfarin, is individual genetic variation or difference in pharmacogenetic genes related to warfarin metabolism. The frequency of these genes defers in different races and populations (22). 

Although the first main problem regarding to its narrow therapeutic index and its interactions has resolved approximately with a strategy calls “maintaining constant weekly dietary vitamin K intake scores” (23). The latter problem, pharmacogenetic polymorphisms, still remained unresolved and even sometimes the role of genetics in adjusting the warfarin dose were ignored (22).

Warfarin which is one of vitamin K antagonist’s medications acts as a vitamin K-epoxide reductase blocker and subsequently prevents formation of vitamin K-dependent clotting factors. Warfarin is primarily metabolized through the P450 system. Thus difference in activity of this system’s isoenzymes such as vitamin K 2, 3-epoxide reductase complex subunit 1 (VKORC1), liver cytochrome P450 polypeptide 9 (CYP2C9), family 2 (CYP4F2), subfamily C, and gamma-glutamyl carboxylase (GGCX) genes can potentially change the INR units (24-28). Among these, two most important single nucleotide polymorphisms (SNPs) in almost all populations are VKORC1 and CYP2C9 (29-34). CYP2C9 is the first identified and most important gene of the P450 system that affects warfarin sensitivity by metabolizing warfarin to inactive metabolites. Up to now, several single nucleotide polymorphisms (SNPs) of CYP2C9 have been identified and finding of these SNPs still continues. These SNPs specially 2C9*2 and 2C9*3 variants affect enzyme activity so that the patients with these variant alleles require less warfarin dose but are more susceptible to bleeding than patients with the wild-type genes (27, 35, 36). CYP4F2 is one of P450 system which is more recently identified with an SNP in rs2108622-1347 C> T (1, 5). However, there is not a consensus on the impact of this SNP on warfarin dosage especially when combined to other variables such as age, race, or when compared to other polymorphisms (4, 37). So, CYP4F2 polymorphism role in warfarin dosage is a new issue in warfarin-therapy related researches (38).

As mentioned above, there are gene polymorphisms differences based on race or ethnic that can affect warfarin dose requirements (39). Several studies in Asia and Iran have been done on the relationship of CYP2C9 and VKORC1 polymorphisms in warfarin dosage (40-42) but no study has been done on CYP4F2 in Iran. To address this issue, the present study examined the relationship between CYP4F2 rs2108622 C>T polymorphisms and warfarin dosage in Iranian patients, Fars Province, who suffer different cardiovascular related conditions requiring warfarin therapy.

## Experimental

*Study population*

Adult patients suffering from different cardiovascular related diseases and with warfarin indication were assessed for enrollment in the study during 2010 to 2012 years. These cardiovascular diseases were including pulmonary thromboendarterectomy (PTE), transient ischemic attack (TIA), rheumatic heart disease (RHD), permanent pacemaker (PPM), cerebrovascular accident (CVA), left atrial (LA) clot, atrial/aortic valve replacement (AVR), mitral valve replacement (MVR), deep vein thrombosis (DVT), percutaneous coronary intervention (PCI), coronary artery bypass grafting (CABG), atrial fibrillation (AF), percutaneous transvenous mitral commissurotomy (PTMC ) and some with combined conditions. All the participants were from Fars province of Iran and were managed in Motahari and Shahid Faghihi anticoagulant clinics belonging to Shiraz University of Medical Science (Shiraz, Fars, Iran). Of the 230 participants, Minimum and maximum duration of warfarin therapy was 3 months to 25 years with an average of 4.7 years. Prior to participation, all subjects were instructed by a specialized nurse for food and drug interactions. Also full guide booklets designed for the study containing information about warfarin were rendered to the participants.

*Inclusion and exclusion criteria*

Inclusion criteria were age over 18 years, Mean INR about 1.5 - 3.5, warfarin indication at least for 2 months and also patients who reached to appropriate INR levels with two INR measurements within one month time period. Thus, the study sample was comprised of patients with established warfarin dose requirements. 

Exclusion criteria were as follows: Pregnant and breast feeding women, injection or consumption of vitamin K, fresh frozen plasma (FFP) and prothrombin compounds, taking simultaneously medications such as Rifampin, Amiodarone, Gemfibrozil, Allopurinol, Fluconazole, Phenobarbital, Carbamazepine and Methimazole 3 weeks before the study, and also diseases that had impact on warfarin standard dose such as kidney failure (CR more than 2.5) and liver cirrhosis. 

*Collection of patient information*

Some critical information required for the study were collected from patients: general information such as name, gender, age, weight, admission number, contact information, drug status including warfarin dose, warfarin duration time, taking other simultaneous medications, cause and indication of warfarin and any history of other complications impact on warfarin dose. Informed consent approved by the institutional review board (Department Of Medical Ethics And Philosophy Of Health, Shiraz University of medical science, Shiraz, Fars, Iran) were used for each person prior to study.

*Study groups and double blinded tests*

The subjects were divided into two groups. Group A (152 subjects) were patients with average daily dose less than 5 mg of warfarin and group B (74 cases) were patients with warfarin average daily dose equal or more than 5 mg. Of these patients, 4 subjects because of not reaching to appropriate therapeutic level of medication were removed from the study. After taking all blood samples and other data, the downstream works such as DNA extraction, PCR, and sequencing were done with another specialized person anonymously.

*Collection of blood samples and Measuring INR levels*

For checking INR level one sterile tube specific for INR measurements containing sodium citrate was used. 2 ml of peripheral blood was collected within 1 day after taking medication. INR measurements were done two times every one week before initiation of the study and one time during the study at Motahari/ Shahid Faghihi anticoagulant clinic (Shiraz University of Medical Science, Shiraz, Iran). INR measurement was done using a Sysmex CA-7000 (Sysmex, Kobe, Japan) automated coagulation analyzer with Innovin (Dade Behring, Marburg, Germany). 

*DNA extraction and Amplification of the target gene and DNA sequencing*

10 ml peripheral blood was taken from the patients and poured into sterile tubes containing EDTA and stored in 4 °C until leukocyte preparation. Red blood cell free leukocytes was stored at -20 °C until all specimens were completed. Genomic DNA was isolated from the leukocyte using Qiagen DNA extraction kit (Qiagen, Valencia, CA, USA). For quantitative estimation of total extracted DNA, absorbance value at 260 nm was measured using UV visible spectrophotometer (spectrophotometry CT5000, USA). 

For amplification of target gene, CYP4F2 V433M (1347 C>T) polymorphism and DNA samples were amplified using polymerase chain reaction (PCR) with the following reaction system: 5 μL of 10-fold concentrated PCR buffer; 1 μL dNTPs (10 mm each); 80 ng template (genomic DNA); 10 pmol each primers; 1 U Taq DNA polymerase. 

Forward and reverse PCR primer sequences were as follows: F 5′- CGGAACTTGGACCATCTACA-3′,R 5′-CCTACTCTCCCACAGGCATTA-3′(43). The PCR reaction mixture was heated to 95 °C for 5 min and then subjected to 35 cycles into which every cycle was done as below: 95 °C (30 seconds), 55 °C (30 seconds). 72 °C (45 seconds). The PCR product was maintained in final extension at 72 °C for 2 min and then cooled to 4 °C. Sequencing of the PCR products identified the following sequences for CYP4F2 rs2108622: CCCCGCACCTCAGGGTCCGGCCACA [C/T] AGCTGGGTTGTGATGGGTTCCGAA. DNA sequencing was done by Bigdye Terminator v3.1 Cycle Sequencing and C300 ABI Prism Genetic analyzer. 

*Statistical methods*

The data were analyzed using SPSS 17.0 (SPSS, Inc., Chicago, IL, USA). Statistical analysis between two independent groups was done using Independent sample t-test. Continuous variables were presented as means and standard deviation, and were compared using ANOVA followed by Bonferroni correction. All data are represented by the mean ± standard deviation. Categorical variables were summarized with frequencies and percentages, and compared using χ2 tests. A value of P < 0.05 was considered statistically significant.

## Results

*Patient characteristics*

Total numbers of male and female participants were 91 (40.3%) and 136 (59.7%). All the patients were from Fars province. The diseases of participants were PTE, TIA, RHD, PPM, CVA, LA clot, AVR, MVR, DVT, PCI, CABG, AF, PTMC and some with combined conditions. Of the 230 participants 226 cases were selected in which 152 subjects were classified into group A (patients with warfarin dose˃5mg/day) and group B including 74 cases (patients with warfarin dose ≥5mg/day). The mean duration of warfarin therapy for group A and B were 6.14 years and 4.29 years, respectively. The mean age for group A and B were 59.9 ± 11.5 and 57.2 ± 12.59 years, respectively. The sex of each group is summarized in Table 4-1.

**Table 4.1:** Sex abundance among groups A and B

**G** **roup**	**Sex**		**p- value**
	**Female** ** Number (%)**	**Male** ** Number (%)**	
**A**	85 (60.6%)	67(39.4%)	0.09
**B**	50(57.1%)	24(42.9%)	

**Table 4.2:** Comparison of the abundance of different rs2108622 SNP genotype in patients

**Genotype**	**rs2108622**			**Total**
**Groups**	**C/T** **Number (%)**	**C/C** **Number (%)**	**T/T** **Number (%)**	
Group A	44 28.9%	89 58.6%	19 12.5%	152
Group B	20 27%	31 41.9%	23 31.1%	74
TOTAL	64 28.3%	120 53.1%	42 18.6%	226
P-value	0.003			

**Diagram 4.1:**
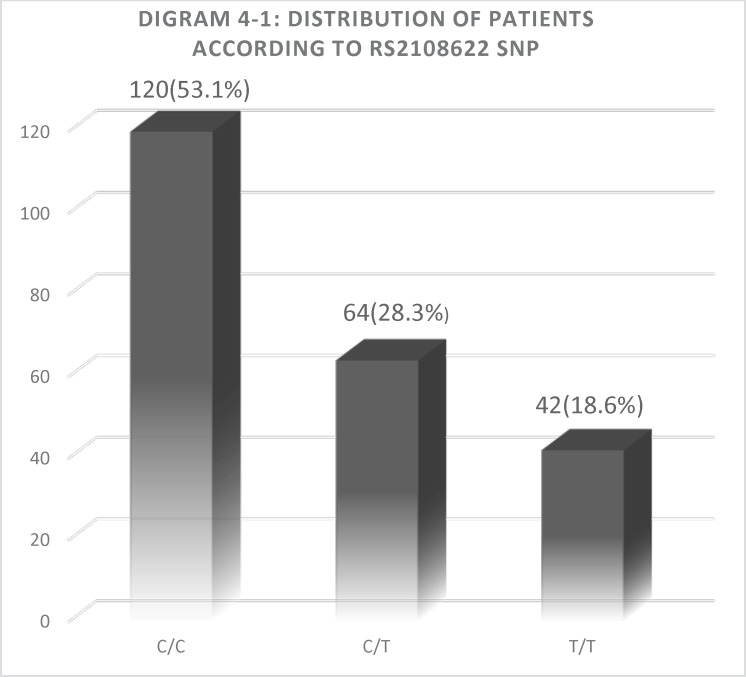
distribution of three genotypes in all participants

**Diagram 4.2:**
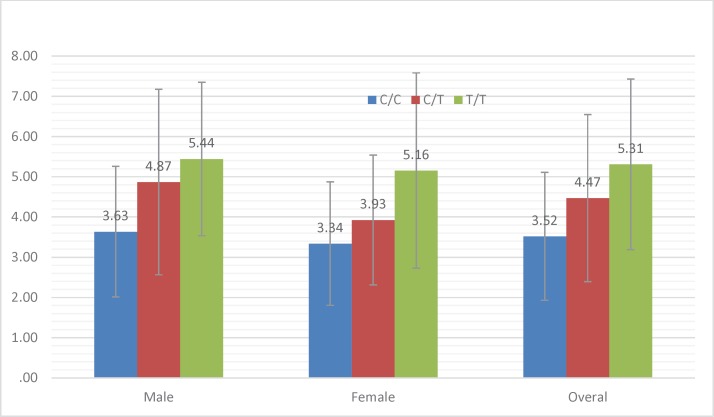
The effect of CYP4F2 polymorphism on the male, female and overall patients’ warfarin dosage

*Daily and weekly dose of warfarin and INR condition*

The first INR measurement was done one month before obtaining genomic DNA and two times during the study. Then, the patients who reached to appropriate INR levels were selected for the study. Min and Maximum therapeutic daily and weekly warfarin dose was 1.07 to 14.28 mg and 7.5 to 100 mg, respectively. The mean therapeutic warfarin dose among all patients was 4.13 mg/Day and 29.11 mg/week. Overall, among all the patients, daily and weekly therapeutic dose was between 1.07 and 100 mg into which 28.2% had therapeutic warfarin dose requirements of less than 20 mg/week; 63.2% were between 20 and 50 mg/week and 8.5% were >50 mg/week. Comparing warfarin dose requirement according to different genotypes and sex difference are shown in diagram 4-2.

Re*lation between warfarin dosage and age*

In our study the mean age of participants were 55.3 years old with minimum and maximum age of 13 and 85 years old, respectively. 5.7% of subjects were between 13-30 years old, 64.8% between 31-60 years old and 29.5% > 60 years old. For investigating the relation between age and warfarin dose, all the participants were divided into two groups including patients >60 years old and ≤ 60 years old. Then the correlation was assessed by Independent Sample T Test. Pearson correlation analysis between age and warfarin dose requirement was -0.29 that was significant (p-value = >0.001). So the results showed that warfarin dose requirements decrease with age.

*Relation between warfarin dosage and sex*

The relation between warfarin dose and sex of subjects was assessed using chi square statistical analysis. There was not a significant difference between case and control groups. (P= 0.754).

*SNP abundance between two groups*

Among all the participants included in the present study, 120 patients (53.1%) had a CYP4F2 wild-type homozygous CC genotype, 64 patients (28.3%) had a heterozygous CT, and 42 patients (18.6%) were homozygous for the mutant TT genotype. The overall frequency of major C allele 50.1%, and the frequency of minor T allele was 40.9%. The frequency of T allele in case and control group was 58.1% and 41.4%,respectively. Also, the abundance of TT, CC, and CT allele in Group B was 23 people (31.1%), 31 people (41.9%) and 20 people (27%), respectively. Abundance of TT, CC, and CT allele in Group A was 19 people (12.5%), 89 people (58.6%) and 44 people (28.9%), respectively. Results of Chi-Square analysis in group B showed a significant difference between rs2108622 SNP and warfarin dose. (P= 0.003). The results are summarized in Table 4-2.

As seen in the Table, there is a significant correlation between T allele prevalence and increased warfarin dose requirements. So that T allele prevalence in control group is 41.4% while in case group it is 58.1%. Also, the ANOVA results showed a very significant correlation between three TT, CC and CT genotype and also mean warfarin dose. (P < 0.001). Average warfarin dose in mutant genotype TT allele, wild-type genotype CC allele and heterozygote CT allele was calculate 5.3 mg, 4.5 mg, and 3.5 mg, respectively. The results are summarized in diagram 4-1 and 4-2.

## Discussion

Warfarin therapy needs to be monitored frequently because of narrow therapeutic window and inter-ethnic or inter individual differences. These differences may rise from factors such as age, sex, diet, diseases, simultaneous medications, and pharmacogentic status. In the present study, on patients referred to Shiraz anticoagulant clinics, the correlation between CYP4F2 SNP and warfarin dose was investigated. Before participation in the study, inclusion and exclusion criteria were checked. Among the 230 participants, 4 patients were removed from the study because of not reaching to proper INR level.

Cardiovascular related diseases in our study were PTE, TIA, RHD, PPM, CVA, LA clot, AVR, MVR, DVT, PCI, CABG, AF, PTMC, and sever MS and some with combined conditions. In other studies regarding warfarin pharmacogenetics, just one or two types of disease were selected for the study; for example Jie-Hue Li et all just focused on valve replacement surgery (38) and Marianne K. Kringen focused on myocardial infarction(4). Most of authors used all the patients referred to anticoagulant clinic centers (1, 44-46). We also used a wider spectrum of cardiovascular diseases. While it has several benefits for checking this SNP in more cardiovascular diseases; however, one of the limitation is low statistical society for checking independent analysis for each disease and then comparing it with total diseases.

Of the 226 cardiovascular related patients participated in the study, 42 patients had a CYP4F2 mutant TT genotype, 64 patients had a heterozygous CT genotype and 120 patients had a wild-type homozygous CC genotype. While the overall frequency of minor T allele was 40.9% however, its frequency in case and control group was 58.1% and 41.4%, respectively. This results of mutant and wild-type allele distributions are approximately similar to that of reported by other authors (1, 4, 38). In all these studies, wild-type CC allele is the most abundant genotype and mutant TT allele is the less abundant genotype. One of the advantages of our study is using different cases of cardiovascular diseases. With these wide ranges of selected diseases, however, our results obey the Harrdi weinburg equilibrium. The results of our study indicate the warfarin dose requirement is increased significantly in Iranian individuals who have at least one T allele, compared to those homozygous for the C allele. 

In our study, the relation between age and warfarin dose was significant and patients > 60 years old had about 1mg warfarin dose requirement less than those ≤ 60 years old. But in the study by Jie-Hui Li (38), he did not find a correlation between age and warfarin dose. It may be because of the duration time of warfarin therapy used in our study. The range of standard INR for patients is about 1.8 ≤ INR ≤ 2.5, but according to the European Society of Cardiology (ESC) guidelines(47). this amount for valve replacement become higher to about 2.5- 3.5. In our study, because of heterogeneity of patients, the INR range was selected between 1.5- 3.5. 

The correlation between Cytochrome P450 iso-enzymes and warfarin dosage are investigated in many studies. Among these, most authors have checked the CYP2C9 and VKORC1 effects on warfarin metabolism while others assessed the combination of these iso enzymes or less the CYP4F2. There are a growing number of studies regarding to the effects of genes on warfarin efficacy (29-31, 40). Some authors selected a combination of Cytochrome P450 iso-enzyme polymorphisms related to warfarin metabolism (37, 44). Some of studies just focused on cyp4f2 polymorphism as a minor significant genetic factor and its effect on warfarin dosage (1, 38, 43, 45, 48, 49). As the ethnic has an impact on warfarin metabolism, the present study was done for the first time in Iranian populations with different cardiovascular related diseases to check CYP4F2 SNP.

One of the limitations of such studies using a wide range of diseases, duration of warfarin therapy, and age of patients participated in the study is that it may have an impact on the statistical analysis methods. Almost, all of the participants were from Fars ethnics. Further studies on Iranian different populations such as Turkish, Kurdish, etc. are suggested to be done. In these studies, it is better to select one disease and narrow age or more diseases with proper statistical numbers. However, with these limitations we had a significant correlation between age and SNP with warfarin dose. 

As FDA emphasizes on this issue that patients with warfarin therapy would better to adjust the dose of medication based on genetic differences (38), many studies have been done in this regard. One of these comprehensive studies is an international study by the International Warfarin Pharmacogenetics Consortium (IWPC) that recently reported a global warfarin dosing algorithm using genetic and clinical information from 5700 patients over nine countries(50). Thus, it seems that in the near future the effect of genetic polymorphisms on warfarin metabolism become established as an important factor for initiation of the therapy. In some studies, the effects of age on warfarin dosage are greater than this SNP genotype (45). The cost of such genotyping tests is another problem for patients. 

## Conclusion

While there are many studies regarding to relation of age and warfarin dose, however there are different results about pharmacogentic status and warfarin dose in different ethnics. The present study was done for the first time on 226 cardiovascular related patients from Fars ethnic of southern Iran. The results of our study demonstrates that polymorphism in the rs2108622 of CYP4F2 gene has a significant impact on the warfarin dose requirement. As there are different ethnics in Iran, further studies of other ethnics are suggested. While there are slightly different results about the effect of this gene SNP on warfarin metabolism, however it seems that in the near future the effects of this gene become more clear. So the cost benefit of such test for patients should be considered in further studies. Finally, it must be noted that finding simple and inexpensive genotyping methods of this SNP would be an effective device for physicians to determine the initiation dose for Iranian patients with warfarin indications.
